# Measures of comorbid cardiometabolic burden and cardiovascular disease risk in people with MRI-confirmed steatotic liver disease: a prospective cohort study

**DOI:** 10.1186/s12933-026-03088-1

**Published:** 2026-01-29

**Authors:** Qi Feng, Pinelopi Manousou, Chioma N. Izzi-Engbeaya, Mark Woodward

**Affiliations:** 1 The George Institute for Global Health (UK), School of Public Health, Faculty of Medicine, Imperial College London, Scale Space, 58 Wood Lane, London, W12 7RZ UK; 2 Division of Digestive Diseases, Department of Metabolism, Digestion and Reproduction, Faculty of Medicine, Imperial College London, London, UK; 3 Department of Hepatology, St Mary’s Hospital, Imperial College Healthcare NHS Trust, London, UK; 4 Section of Investigative Medicine and Endocrinology, Department of Metabolism, Digestion and Reproduction, Faculty of Medicine, Imperial College London, London, UK; 5 Department of Endocrinology, St Mary’s Hospital, Imperial College Healthcare NHS Trust, London, UK; 6 The George Institute for Global Health (Australia), University of South New Wales, Sydney, Australia

**Keywords:** Steatotic liver disease, Cardiometabolic risk factor, Cardiovascular disease

## Abstract

**Background:**

Steatotic liver disease (SLD) is commonly associated with higher burden of cardiometabolic risk factors (CMRFs). This study aimed to examine the associations between CMRF count, patterns and risk of cardiovascular disease.

**Methods:**

We included 10,121 UK Biobank participants (39% women) with MRI-confirmed liver steatosis. Latent class analysis was used to derive CMRF patterns based on 5 CMRFs (obesity, diabetes, hypertension, high triglycerides and low HDL). Cox models were used to estimate associations between CMRF count and patterns with incidence and mortality of cardiovascular disease (CVD), and all-cause mortality.

**Results:**

Approximately 95% of SLD participants had ≥ 2 CMRFs. During a median follow-up of 4.9 years, 268 CVD events and 212 deaths were recorded. Higher CMRF count was independently associated with elevated risk of CVD (HR per each additional CMRF: 1.23 (1.08, 1.40)), CVD mortality (1.47 (1.07, 2.02)), and all-cause mortality (1.25 (1.08, 1.44)). Three distinct CMRF patterns were identified, reflecting varying levels of CMRF burden and demographic characteristics. While certain patterns with high CMRF burden were associated with increased CVD risk, the associations were substantially attenuated after adjusting for CMRF count.

**Conclusions:**

CMRF burden is a key determinant of cardiovascular risk in people with SLD, but data-driven CMRF patterns do not improve risk prediction beyond simple counts. CMRF count remains a practical measure of cardiometabolic burden.

**Supplementary Information:**

The online version contains supplementary material available at 10.1186/s12933-026-03088-1.

## **Research insights**

**What is currently known about this topic?**

- Cardiometabolic risk factors (CMRFs) increase the risk of cardiovascular diseases (CVD) in people with steatotic liver disease (SLD).

**What is the key research question?**

- How do CMRF patterns compare with CMRF count, regarding their associations with CVD risk in people with SLD?

**What is new?**

- Each additional CMRF increased CVD risk by 23%.
- Three distinct CMRF patterns were identified using latent class analysis.
- Although certain CMRF patterns increased CVD risk, the associationsdisappeared after adjusting for CMRF count.

**How might this study influence clinical practice?**

- Complex CMRF patterns do not improve CVD risk prediction beyondsimple counts. CMRF count remains an effective and pragmatic measurefor cardiometabolic burden.

## Background

Steatotic liver disease (SLD) is the most common chronic liver condition, encompassing three subtypes: metabolic dysfunction-associated steatotic liver disease (MASLD), alcohol-related liver disease (ALD), and metabolic-alcohol-related liver disease (MetALD) [1, 2]. It is strongly associated with cardiovascular disease (CVD), and premature mortality [3–5]. Comorbid cardiometabolic risk factors (CMRFs), such as diabetes, hypertension, obesity and dyslipidemia, are highly common in people with SLD [6]. Accumulating evidence suggests the CMRF burden has been an important driver for complications and mortality in the SLD population [6].

However, the optimal way to measure cardiometabolic burden remains unclear. The latest definition employed a threshold-based approach (presence vs. absence) [7], which overlooks the heterogeneity and clustering of CMRFs. Prior studies have primarily examined the individual or additive effects of CMRFs, typically using a count-based approach, on outcomes such as liver steatosis [8], fibrosis [9, 10], cardiovascular disease (CVD) and mortality [11–15]. While practical, this method may not adequately reflect the complex interplay between them.

Emerging evidence suggests that distinct patterns or clusters of cardiometabolic abnormalities may confer differential risks of adverse outcomes. For example, a study by Chung et al. divided MASLD population into diabetic, overweight and lean subtypes and found that diabetic MASLD was associated with the highest mortality [16]. However, this classification was based on predefined clinical categories. It remains unclear whether it represents the most meaningful way to capture CMRF patterns. Little is known about how CMRFs cluster in a data-driven way in the SLD population, and whether such derived clusters provide more value in prognosis prediction.

Therefore, this study aimed to identify data-driven CMRF patterns, and to examine the associations between CMRF count, patterns and CVD risk and mortality, and to compare the predictive value of CMRF count and patterns among individuals with SLD.

## Methods

### Participants

The UK Biobank is a large cohort of approximately half million individuals. At baseline assessment between 2006 and 2010, data were collected on sociodemographic background, lifestyle, environmental exposures, physical measures and medical history. Biological samples of blood, urine and saliva were also collected [17]. Between 2010 and 2013, a subset of ~ 20,000 participants underwent a repeat assessment. Since 2014, participants have been invited to attend imaging visits, where magnetic resonance imaging (MRI) scans of the brain, heart and abdominal organs were conducted. As of June 2025, liver MRI data are available for ~ 40,000 participants [18]. Baseline data were updated during repeat and imaging visits. Biochemistry data were available at baseline and repeat assessments. All participants were followed up via linkage to national death registries, cancer registries and hospital admission records.

Liver MRI scans were acquired using a Siemens MAGNETOM Aera1.5T scanner (Syngo MR D13) and the LiverMultiScan protocol from Perspectum Ltd (UK) [19]. Liver fat content was quantified using proton density fat fraction (PDFF), with PDFF ≥ 5% indicating presence of liver steatosis.

### CMRF and CMRF patterns

We examined five CMRFs, in line with the updated SLD nomenclature framework [7]:

- Overweight/obesity: body mass index (BMI) ≥ 25 kg/m² and/or waist circumference > 94 cm (male) (> 80 cm (female));
- Prediabetes/diabetes: glycated haemoglobin (HbA1c) ≥ 39 mmol/mol and/or diagnosis of type 2 diabetes and/or on treatment for type 2 diabetes;
- Hypertension: systolic blood pressure (BP) ≥ 130 and/or diastolic BP ≥ 85 mmHg and/or on antihypertensive drug treatment or diagnosis of hypertension;
- High triglycerides (TG): plasma TG ≥ 1.70 mmol/L and/or on lipid lowering treatment;
- Low high-density lipoprotein (HDL) cholesterol: HDL-cholesterol ≤ 1.0 mmol/L (male) (≤ 1.3 mmol/L (female)) and/or on lipid lowering treatment.

CMRF status was determined using survey data from all three assessment visits and biomarker data from the first two assessments, if available. Biomarker data from imaging visits were not available.

We applied latent class analysis (LCA) to derive distinct CMRF patterns among individuals with SLD [5]. Fundamentally, LCA assumes that the joint distribution of the observed variables can be explained by a latent categorical variable with K classes, with each class having its own set of conditional probabilities. LCA uses maximum likelihood estimation via the expectation–maximization algorithm to calculate, for each individual, the posterior probability of belonging to each class, as well as the probability of each variable given class membership. The analysis proceeded in two main steps: first, we compared models with increasing numbers of clusters and selected the model with the lowest Bayesian Information Criterion (BIC) as the optimal solution. BIC has been used as the standard criterion in choosing the best-fitting LCA models, while penalising overfitting [5]. Second, we characterized each cluster based on the prevalence of individual CMRFs. A binary matrix indicating the presence of each CMRF was used as model input. We evaluated models with 2 to 12 clusters, and selected the optimal number of clusters based on the lowest value of the sample size-adjusted Bayesian Information criterion (aBIC). Each participant was assigned to one of the mutually exclusive clusters.

As a sensitivity analysis, we also examined three predefined CMRF patterns [16]:

- Diabetic SLD: SLD with prediabetes/diabetes, regardless of other CMRFs.
- Overweight SLD: SLD without prediabetes/diabetes, but with overweight/obesity, regardless of other CMRFs.
- Lean SLD: SLD with neither prediabetes/diabetes nor overweight/obesity, regardless of other CMRFs.

### Outcomes

The primary outcomes were incident CVD, and the secondary outcomes were incidences of myocardial infarction (MI) and stroke, all-cause mortality, and CVD-specific mortality. Outcomes were ascertained through death registries and hospital admission records, using the following the International Classification of Disease, 10th revision 10 (ICD-10) codelists: I22-I23, I24.1 and I25.2 for MI; and I60-61 and I63-64 for stroke, respectively. These codelists have been used previously [20, 21]. Participants were censored at the date of outcome diagnosis, death, or the last day of follow-up (30 October 2022), whichever occurred first. Participants with prevalent CVD prior to the imaging visit were excluded from the incidence analysis.

### Covariates

Ethnicity was classified into White, Asian, Black, and mixed/others. The Townsend Deprivation Index is a postcode-based measure of socioeconomic status. Educational attainment was categorised as below secondary, lower secondary, upper secondary, vocational training, and higher education. Smoking status was self-reported as current, previous or never smoking. Alcohol consumption was assessed via self-reported intake of various alcoholic drinks; the consumption was summed up to derive average daily alcohol consumption (g/d). Dietary factors were assessed using a food frequency questionnaire, and consumptions of vegetable, red meat (including pork, mutton and beef) and processed meat were summarised. Physical activity level was measured with the International Physical Activity Questionnaire; individuals were categorized into low, moderate and high levels, based on the frequency, duration and intensity of their physical activities. Systolic and diastolic blood pressures were measured twice, and the averages were used. Blood biochemistry markers were measured at a central laboratory. Uses of antihypertensive drugs, statins and antidiabetic drugs were self-reported. SLD subtypes (MASLD, MetALD and ALD) were defined using the latest SLD nomenclature framework, based on presence of CMRFs and average daily alcohol consumption level [7]. All these variables were obtained during the UK Biobank imaging visit, at the same as the liver MRI, except for blood biochemistry, which was measured at the UK Biobank baseline visit and/or repeat assessment visit, using the later measurement when available.

### Statistical analysis

We excluded people who had withdrawn consent to participate, those with missing data on liver MRI data or CMRFs, those with PDFF < 5% (absence of liver steatosis) and those with diagnosis of other chronic liver conditions (viral hepatitis, liver fibrosis, liver cirrhosis, hepatocellular carcinoma, hemochromatosis, Wilson’s disease, biliary cirrhosis, autoimmune hepatitis, primary sclerosing cholangitis, toxic liver disease and Budd-Chiari syndrome). For incidence analyses, participants with prevalent CVD were excluded.

The basic characteristics of participants at the imaging visit were summarised across the derived CMRF patterns. The distribution of CMRFs within each cluster was visualised using radar plots, comparing the prevalence of each CMRF within each pattern to the overall SLD.

Cox proportional hazards models were fitted to assess the associations between CMRF count, CMRF patterns and the outcomes of interest, expressed as hazard ratio (HR) and 95% confidence interval (CI). For associations of CMRF count, we fitted the model with CMRF count as continuous variable (per one additional CMRF) in primary analysis and as a categorical variable (1, 2, 3, 4, 5) in sensitivity analysis. Date of imaging visit was used as the time origin. The associations were estimated in three progressively adjusted models. Model 1 was unadjusted. Model 2 were stratified by age groups (< 60, 60–64, 65–69 and ≥ 70), and adjusted for ethnicity, Townsend Deprivation Index, education, smoking, physical activity, average daily alcohol consumption, consumption of vegetable, red meat and processed meat, and uses of statins, antihypertensive and antidiabetic drugs. Model 3 was additionally adjusted for CMRF count, to further delineate the independent associations of CMRF patterns. The proportional hazards assumption was examined by scaled Schoenfeld residuals; no evidence was observed for its violation, except for age, which was included as a stratifying variable in the model to address this issue. To account for competing risks of death in CVD incidence and mortality outcomes, we performed Fine-Gray subdistribution model with the same covariate adjustment as in the Cox models.

We examined the associations stratified by SLD subtypes. We also performed sex-specific analyses to examine effect modification by sex. We additionally examined the independent associations of each individual CMRF, adjusting for all other CMRFs.

## Results

**Fig. 1:**
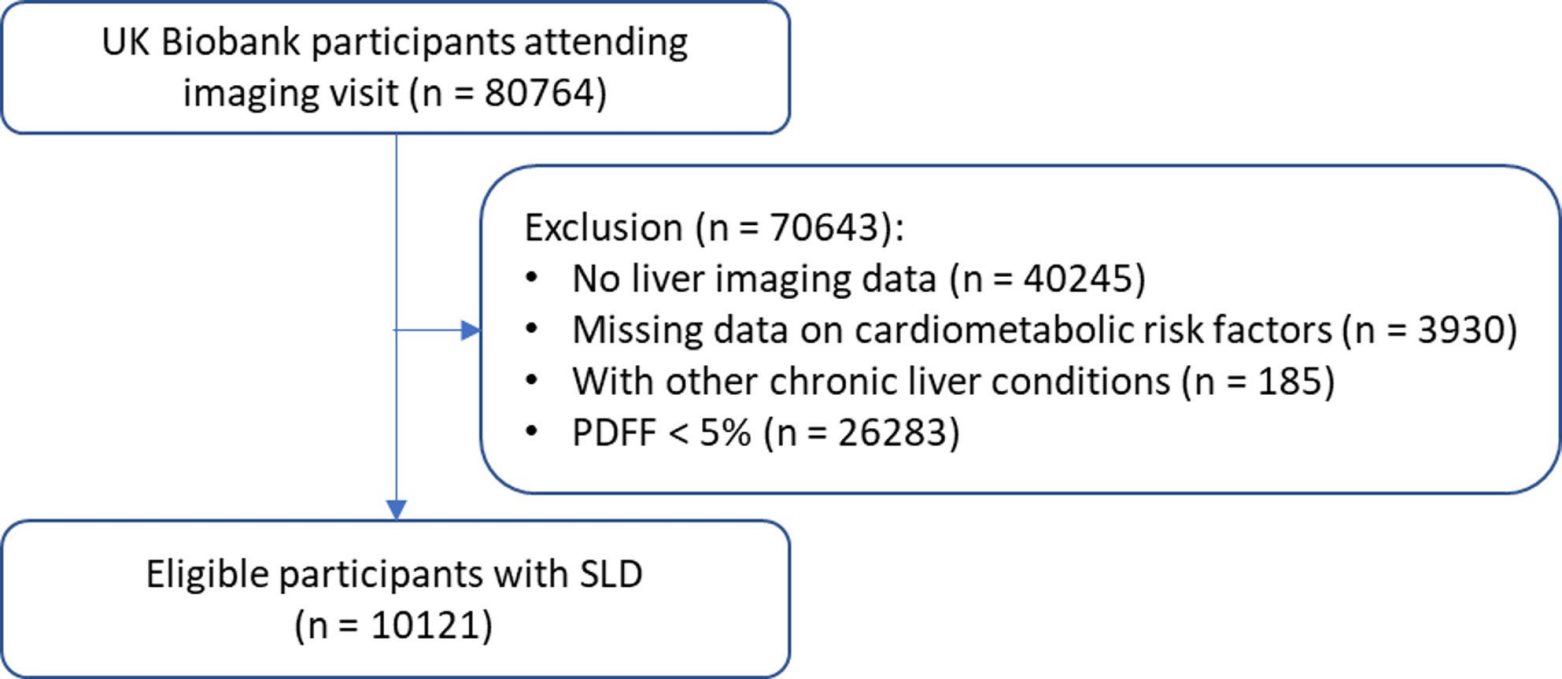
Flowchart of participants selection SLD: steatotic liver disease. PDFF: proton density fat fraction. Other chronic liver conditions include viral hepatitis, hemochromatosis, Wilson’s disease, biliary cirrhosis, autoimmune hepatitis, primary sclerosing cholangitis, drug-induced liver injuries, and Budd-Chiari syndrome

We identified 10,121 participants with SLD (60.9% males, age 64.7 (SD 7.4) years) (Fig. 1). The most common CMRFs were hypertension (prevalence 92.1%), overweight/obesity (91.8%), and high TG (58.6), while 22.3% of participants had prediabetes/diabetes. CMRF burden was generally high: 94.7% SLD people had ≥ 2 CMRFs.

## CMRF patterns

**Fig. 2:**
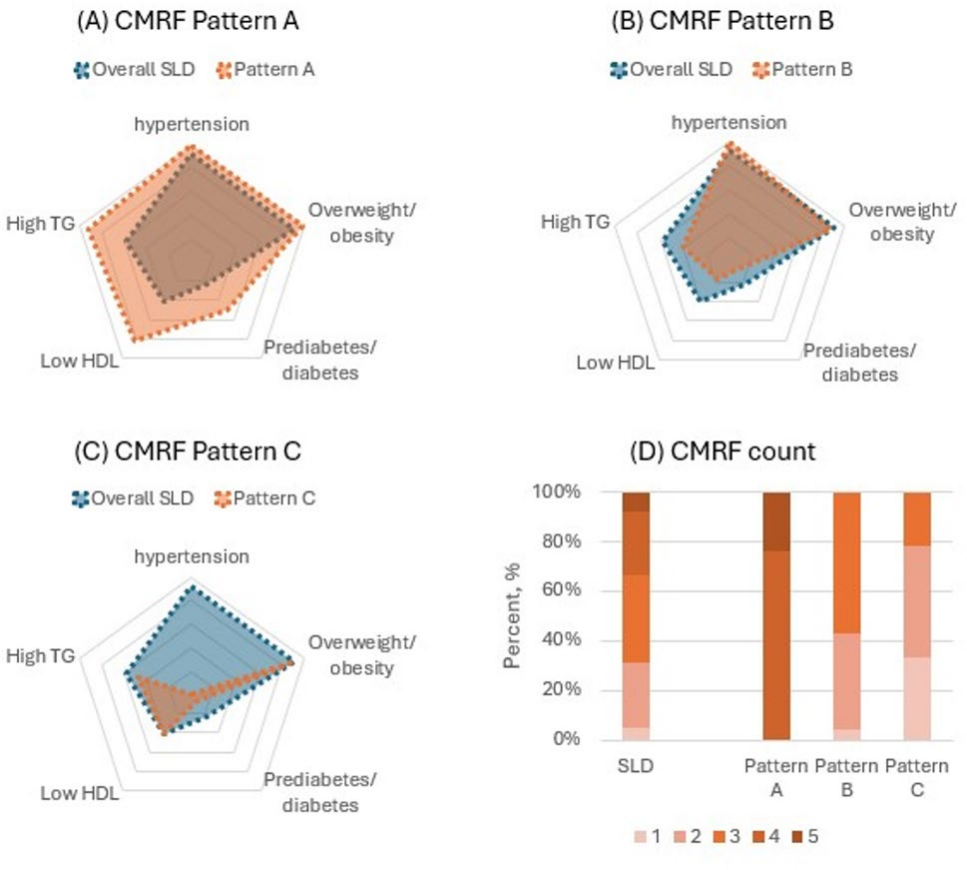
Characteristics of latent class analysis derived cardiometabolic risk factor patterns SLD: steatotic liver disease. TG: triglycerides. HDL: high density lipoprotein cholesterol. CMRF: cardiometabolic risk factor. A-C are radar plots showing the CMRF prevalence within each LCA-derived cluster compared to the overall SLD population

The LCA model with 3 clusters showed the lowest aBIC value (supplementary Fig. 1). Hence three distinct CMRF patterns were identified: 3373 (33.3%) participants were classified as Pattern A, 5985 (59.1%) as Pattern B, and 761 (7.5%) as Pattern C. Pattern A showed higher prevalence of all five CMRFs than the general SLD population, indicating a high cardiometabolic burden, while Pattern B had lower prevalence of dyslipidemia and prediabetes/diabetes. Pattern C was characterised with lower prevalence of hypertension and prediabetes/diabetes, but comparable prevalence of overweight/obesity and dyslipidaemia, than overall SLD participants (Fig. 2). All individuals in Pattern A had ≥ 4 CMRFs, while those in Patterns B and C had ≤ 3 CMRFs. More specifically, 66.6% of Pattern C and 95.4% of Pattern B had ≥ 2 CMRFs.

Pattern C participants were generally younger than others (Table 1). Pattern A participants were more likely to be smokers, living in deprived areas, to have higher BMI and waist circumference, but less likely to consuming alcohol or engage physical activity. In contrast, Pattern B participants had the highest alcohol consumption. Overall, Pattern A resembled the MASLD phenotype, which has lowest alcohol intake but highest CMRF burden. Pattern C had the least CMRF burden, while Pattern B had the highest alcohol consumption and the highest proportion of MetALD and ALD together (26.8%). Supplementary Table 1 presents participant characteristics stratified by CMRF count.

**Table 1:** Baseline characteristics of participants with steatotic liver disease, stratified by latent class analysis-derived cardiometabolic risk factor patterns

	Total	Pattern A	Pattern B	Pattern C
	*n* = 10,121 (100.0%)	*n* = 3375 (33.3%)	*n* = 5985 (59.1%)	*n* = 761 (7.5%)
Sex, male	6163 (60.9%)	1766 (52.3%)	4062 (67.9%)	335 (44.0%)
Age, years	64.7 (7.4)	65.8 (7.2)	64.7 (7.4)	60.4 (7.2)
Townsend Deprivation Index				
1st fifth (least deprived)	1929 (19.1%)	605 (17.9%)	1191 (19.9%)	133 (17.5%)
5th fifth (most deprived)	2154 (21.3%)	800 (23.7%)	1193 (19.9%)	161 (21.2%)
Education, higher education	4274 (42.2%)	1318 (39.1%)	2604 (43.5%)	352 (46.3%)
Ethnicity, White	9792 (96.7%)	3252 (96.4%)	5810 (97.1%)	730 (95.9%)
Smoking, never	5881 (58.1%)	1857 (55.0%)	3541 (59.2%)	483 (63.5%)
Alcohol drinking, g/d*	11.5 (2.6, 25.1)	7.5 (1.3, 19.6)	14.8 (4.6, 28.9)	8.5 (1.7, 20.1)
Physical activity, high	3324 (32.8%)	985 (29.2%)	2097 (35.0%)	242 (31.8%)
BMI, kg/m2	29.7 (4.4)	30.8 (4.5)	29.1 (4.2)	28.5 (3.7)
Waist circumference, cm	97.7 (11.2)	100.1 (11.3)	96.9 (11.0)	93.0 (9.7)
Systolic blood pressure, mmHg	144.3 (17.4)	145.6 (16.9)	146.9 (16.1)	120.4 (6.9)
Diastolic blood pressure, mmHg	82.1 (9.7)	81.5 (9.6)	83.6 (9.5)	73.3 (6.0)
Hypertension	9326 (92.1%)	3341 (99.0%)	5985 (100.0%)	0 (0%)
Overweight/obesity	9287 (91.8%)	3349 (99.2%)	5241 (87.6%)	697 (91.6%)
Prediabetes/diabetes	2256 (22.3%)	1703 (50.5%)	496 (8.3%)	57 (7.5%)
High triglycerides	5929 (58.6%)	3140 (93.0%)	2422 (40.5%)	367 (48.2%)
Low HDL-cholesterol	4064 (40.2%)	2777 (82.3%)	974 (16.3%)	313 (41.1%)
SLD subtypes				
MASLD	7791 (77.0%)	2792 (82.7%)	4381 (73.2%)	618 (81.2%)
MetALD	1838 (18.2%)	469 (13.9%)	1242 (20.8%)	127 (16.7%)
ALD	492 (4.9%)	114 (3.4%)	362 (6.0%)	16 (2.1%)

The table shows count(percent) for categorical variables and mean (standard deviation) for continuous variable, unless specially marked. *: showing median (interquartile interval). All p values < 0.01 for differences between the three groups. P values were derived from chi-square test for categorical variables, analysis of variance test for normally distributed continuous variables, and Kruskal-Wallis test for other continuous variables. BMI: body mass index. HDL: high density lipoprotein. SLD: steatotic liver disease. MASLD: metabolic dysfunction associated steatotic liver disease. MetALD: metabolic and alcohol related liver disease. ALD: alcohol related liver disease

## Associations with outcomes

For CVD incidence analysis, we excluded 300 participants with CVD diagnosed before the imaging visit, resulting in 9,821 participants. During median follow-up of 4.8 years, 268 participants were diagnosed with incident CVD (5.72 per 1000 person-years), including 184 MI and 85 stroke.

During a median follow-up of 4.9 years, 212 people died (4.23 per 1000 person-years), and 46 from CVD. Each additional CMRF was associated with a 23% elevated risk of CVD (HR 1.23 (1.08, 1.40)), 19% elevated risk of MI (1.19 (1.02, 1.40)), 30% elevated risk of stroke (1.30 (1.03, 1.64)). Higher CMRF count was also positively associated with increased all-cause mortality (1.25 (1.08, 1.44)), and CVD mortality (1.47 (1.07, 2.02)). (Table 2)

**Table 2:** Associations between cardiometabolic risk factor number and patterns with all-cause mortality, cardiovascular mortality and risks

		Latent class analysis derived cardiometabolic risk factor patterns		
	Per 1 more risk factor	Pattern A	Pattern B	Pattern C
*Incidence*				
CVD	268 / 9821	105 / 3216	156 / 5855	7 / 750
Model 1	1.18 (1.05, 1.33)	Reference	0.87 (0.68, 1.12)	0.38 (0.17, 0.82)
Model 2	1.23 (1.08, 1.40)	Reference	0.80 (0.61, 1.05)	0.42 (0.19, 0.92)
Model 3	–	Reference	1.24 (0.78, 1.97)	0.77 (0.30, 1.95)
MI	184 / 9821	74 / 3216	106 / 5855	4 / 750
Model 1	1.19 (1.03, 1.37)	Reference	0.85 (0.63, 1.14)	0.31 (0.11, 0.86)
Model 2	1.19 (1.02, 1.40)	Reference	0.80 (0.58, 1.11)	0.39 (0.14, 1.09)
Model 3	--	Reference	1.08 (0.62, 1.89)	0.59 (0.18, 1.96)
Stroke	85 / 9821	31 / 3216	51 / 5855	3 / 750
Model 1	1.17 (0.95, 1.44)	Reference	0.96 (0.62, 1.51)	0.54 (0.16, 1.78)
Model 2	1.30 (1.03, 1.64)	Reference	0.81 (0.50, 1.30)	0.46 (0.14, 1.55)
Model 3	–	Reference	1.70 (0.75, 3.87)	1.29 (0.29, 5.83)
*Mortality*				
All-cause	212 / 10,121	82 / 3375	115 / 5985	15 / 761
Model 1	1.12 (0.99, 1.28)	Reference	0.85 (0.64, 1.13)	1.16 (0.66, 2.02)
Model 2	1.25 (1.08, 1.44)	Reference	0.69 (0.51, 0.92)	1.11 (0.63, 1.97)
Model 3	–	Reference	1.18 (0.70, 1.97)	2.31 (0.98, 5.16)
CVD	46 / 10,121	22 / 3375	24 / 5985	0 / 761
Model 1	1.39 (1.04, 1.85)	Reference	0.66 (0.37, 1.18)	NA
Model 2	1.47 (1.07, 2.02)	Reference	0.58 (0.31, 1.08)	NA
Model 3	–	Reference	1.01 (0.32, 3.16)	NA

CVD: cardiovascular disease. MI: myocardial infarction. Model 1: the cox model was unadjusted. Model 2: the Cox model was stratified by age group, adjusted for sex, ethnicity, education, Townsend Deprivation Index (fifths), physical activity level, average daily alcohol drinking, smoking status, diet (vegetable, processed meat, and red meat) and medication uses (antihypertensives, statins, and antidiabetics). Model 3: model 2 + CMRF count

Using categorical CMRF counts, we also observed log-linear trends (Supplementary Table 2). Compared to individuals with one CMRF, those with 5 CMRFs had more than three times the risk of CVD (HR 3.58 (1.36, 9.43)), especially MI (3.95 (1.16, 13.47)). The association with incident stroke was positive but did not reach statistical significance (2.81 (0.56, 14.08)).

The three derived CMRF patterns showed distinct disease risks (Table 2). Compared to Pattern A (with highest CMRF burdens), Pattern C demonstrated a significantly lower risk of CVD (0.42 (0.19, 0.92)), but a non-significant negative association with MI (0.39 (0.14, 1.09)) and stroke (0.46 (0.14, 1.55)). However, adjusting for CMRF counts attenuated the associations. For example, the residual association between Pattern C and CVD risk was HR 0.77 (0.30, 1.95))

Pattern B also demonstrated generally lower CVD risk than Pattern A (HR 0.80 (0.61, 1.05)), with HR 0.80 (0.58, 1.11) for MI, and 0.81 (0.50, 1.30) for stroke. These associations were attenuated after adjusting for CMRF count.

We observed null evidence for difference in all-cause mortality and CVD-specific mortality across the three CMRF patterns, probably due to small number of events. (Table 2)

Sensitivity analysis using predefined CMRF patterns (lean, overweight and diabetic SLD) generated similar findings. Overall, 6.8%, 70.9% and 22.3% participants had lean, overweight/obese and diabetic SLD. Supplementary Fig. 2 shows the characteristics of these patterns. Compared to the overall SLD cohort, lean SLD participants showed slightly lower prevalence of dyslipidemia and similar prevalence of hypertension. Overweight/obese SLD individuals showed similar prevalence of dyslipidemia and hypertension, but no prediabetes/diabetes. Diabetic SLD showed higher prevalence of all CMRFs. Supplementary Table 3 shows their basic characteristics. Supplementary Table 4 show the associations with the outcomes in the three progressively adjusted models. Compared to lean SLD, individuals with overweight SLD and diabetic SLD showed the trend of progressively elevated risk of CVD, MI and stroke, although most associations did not reach statistical significance, and were attenuated after adjusting for CMRF count. For example, the association of diabetic SLD was attenuated from 1.94 (1.02, 3.70) to 1.45 (0.68, 3.10) for all-cause mortality, and from 1.56 (0.89, 2.72) to 1.06 (0.55, 2.07) for CVD risk.

In Fine-Gray models accounting for competing risk, results were consistent with the primary analysis: CMRF count was associated with the outcomes, while CMRF patterns showed null independent associations after adjusting for CMRF count. (Supplementary Table 5)

### Associations stratified by SLD subtypes

Among the 7791 participants with MASLD, findings were consistent: in the fully adjusted model, CMRF count was associated with mortality and CVD risk, whereas CMRF patterns showed null associations (Supplementary Table 6). Among the 1838 participants with MetALD, CMRF count showed positive but statistically non-significant associations, probably due to small sample size in this subgroup (Supplementary Table 7). We did not conduct parallel analyses in individuals with ALD because of the small sample size (*n* = 492).

#### Sex-specific associations

Each additional CMRF elevated CVD risk by 17% in males (1.17 (1.01, 1.36)), and 42% in females (HR 1.42 (1.08, 1.87)), although the difference was not significant between sexes (ratio of HR: 0.81 (0.60, 1.11)). (Supplementary Table 8).

### Individual CMRFs

After full adjustment for all covariates and all the other CMRFs, diabetes and high TG were significantly associated with higher mortality, with HRs of 1.56 (1.16, 2.09) and 1.34 (1.01, 1.80), respectively. Diabetes also increased CVD risk by 35% (1.35 (1.03, 1.76)). Hypertension showed a positive association with CVD risk (HR 2.11 (0.99, 4.52)). (Supplementary Table 9)

## Discussion

In this cohort of MRI-confirmed SLD participants with CMRFs, 94.8% had ≥ 2 CMRFs and 68.8% ≥ 3 CMRFs. The cardiometabolic burdens are positively associated with CVD incidence, CVD mortality and all-cause mortality. Using LCA, we derived three distinct CMRF patterns reflecting varying degrees of cardiometabolic burdens and lifestyle factors, with Pattern A resembling a high-risk MASLD phenotype. Although certain CMRF patterns demonstrate significantly elevated risk of the outcomes, the associations were substantially attenuated after adjusting for CMRF counts, and the added value of CMRF patterns to CMRF count was minimal regarding predicting prognosis.

Our findings of positive associations between CMRF count and CVD risk and mortality in individuals with SLD are consistent with and extend the growing body of evidence linking cardiometabolic burden with adverse extrahepatic outcomes. Several studies have used CMRF count as a measure for cardiometabolic burden and shown broadly consistent results. Lee et al. [9] found that having ≥ 3 CMRFs was associated with more advanced fibrosis status in people with MASLD and MetALD. Huang et al. [10] also found a positive associations between CMRF count and liver steatosis and fibrosis, independent of alcohol intake.

A near log-linear increase in all-cause mortality by CMRF count was observed by Li et al. [11], that compared to people with a single CMRF, people with 2, 3, 4, and 5 CMRFs showed progressively elevated mortality by 71%, 92%, 116% and 156%, respectively. Similarly, Iwaki et al. [12] reported that presence of CMRF was associated with advanced histological features of liver and higher risk of mortality and liver-related events. Park et al. [13] showed a dose-response relationship between higher CMRF count and overall mortality, and mortality of CVD and liver disease, although their use of non-SLD individuals as reference group may have exaggerated these associations, and made it difficult to distinguish the effects of steatosis and cardiometabolic burden. Pustjans et al. [15] confirmed that 65% of individuals with SLD had ≥ 3 CMRFs, and that higher numbers of CMRFs were associated with liver stiffness and higher mortality.

Beyond evaluating CMRF counts, we explored data-driven CMRF patterns and identified three distinct phenotypes. Pattern C represents a low-risk group, characterised by the lowest prevalence of all five CMRFs, particularly a near absence of hypertension and diabetes, as well as younger age, higher educational attainment, healthier lifestyle behaviour, and a greater proportion of females. By contrast, Pattern A represents the highest cardiometabolic burden, with the highest prevalences of all CMRFs, reflecting a MASLD-dominant phenotype within the SLD spectrum. This group also has older age, lower socioeconomic status, lower education attainment, the lowest alcohol consumption, and physical activity. Pattern B exhibited an intermediate cardiovascular burden overall, but a unique profile: all individuals had hypertension, yet the prevalence of diabetes and dyslipidemia was relatively low. Pattern B has a substantially higher proportion of MetALD and ALD together, accompanied by highest alcohol consumption. Briefly, pattern A represents older individuals with high overall cardiometabolic burden, particularly diabetes. Pattern B represents older individuals with highest alcohol intake and cardiometabolic burden dominated by hypertension. Pattern C represents younger individuals with low cardiometabolic burden. Notably, these patterns were associated with distinct outcome risks. Compared to Pattern C, Pattern A showed higher CVD risk and mortality. Pattern B, despite its unique hypertension-dominant profile, did not show significantly elevated risk, likely due to smaller sample size and fewer outcome events. Although Patterns A and B showed different CMRF and lifestyle profiles, their differences regarding CVD risk were statistically non-significant, which could be due to limited number of outcome events during follow-up in our study. Therefore, future studies with larger sample size and longer follow-up are warranted. Patients in both Pattern A (metabolic-dominant) and Pattern B (hypertension/high alcohol–dominant) may benefit from aggressive cardiometabolic risk management, and diabetes, dyslipidemia, and hypertension remain key therapeutic targets. Alcohol reduction is especially relevant for Pattern B.

Certain CMRF patterns were significantly associated with progression to complications and mortality, suggesting they may capture clinically relevant risk subgroups. However, these associations were largely attenuated after adjusting for CMRF count, likely because the high-risk patterns reflected a greater number of CMRFs, which captures the elevated risk. The LCA-derived patterns also do not map neatly onto clinically recognisable phenotypes, and, therefore, are not intended to guide clinical decision-making. Taken together, these findings suggest that while CMRF patterns provide interesting risk stratification, the simple count of CMRFs remains a robust and practical measure of cardiometabolic burden, which clinicians can readily apply to people with SLD.

In examining the effects of individual CMRFs, hypertension showed the highest HR point estimate, although with a wide CI, diabetes showed a significantly strong association. Although Pattern B had universal hypertension, it also had substantially lower prevalence of diabetes and dyslipidemia compared with Pattern A. Although hypertension showed the highest HR point estimate for CVD risk among all individual CMRFs, the joint burden of diabetes and dyslipidemia may outweigh the effect of hypertension alone. This may explain why Pattern B, despite universal hypertension, did not exhibit higher CVD risk than pattern A, which had a markedly higher prevalence of diabetes and dyslipidemia and a greater overall cardiovascular burden.

Our findings highlight the role of cardiometabolic burden in shaping extrahepatic outcomes in individuals with SLD, particularly in relation to CVD and mortality. From a clinical point of view, simple CMRF count offers a practical, easily implementable tool for risk stratification in SLD, without requiring complex clustering approaches. The strong associations between cardiometabolic burden and CVD risk highlights the need for multidisciplinary management involving hepatology, cardiology, endocrinology and primary care. Identifying individuals with higher CMRF counts may support earlier intervention and targeted cardiovascular prevention in SLD. From a public health point of view, given the high prevalence of SLD and the near-universal presence of 2 or more CMRFs in this population, cardiometabolic burden represents a significant and modifiable driver of CVD risk. Our findings reinforce the importance of population-level strategies promoting cardiometabolic health, including obesity prevention, diabetes control, lipids and hypertension management, to reduce cardiovascular complications of SLD. The simplicity of CMRF count also facilitates incorporation into routine screening and risk communication in both clinical practice and public health programmes.

This study has several strengths. We used a large MRI-confirmed SLD cohort with comprehensive cardiometabolic phenotyping and prospective follow-up. This is also the first study to provide empirical evidence on comparison between CMRF counts and data-driven CMRF patterns regarding their associated CVD risks, which is one of the main drivers in mortality in people with SLD. We also acknowledge some limitations. First, the median follow-up duration was relatively short (4.9 years) and the numbers of events was limited, which may weaken the stability of multivariable estimates and limit the ability to detect association for less common outcomes, particularly those reflecting long-term CVD and mortality risks. This limitation was further amplified in subgroup analyses stratified by sex or SLD subtype, which was why we restricted subgroup analyses to these two key variables only. Second, the cohort’s mean age was 64 years, and the findings may not be generalisable to younger individuals with SLD, who may exhibit different risk profiles and disease trajectory. Third, UK Biobank cohort is affected by healthy volunteer bias and is not completely representative of the general UK population, regarding ethnicity, socioeconomic status and health status. This limits the external validity of our results, particularly in underrepresented groups. Fourth, although we found that CMRF count is a pragmatically effective measure for cardiometabolic burden in people with SLD, future research is warranted to explore the development and validation of a weighted CMRF score based on the relative contribution of each factor. Fifth, some variables, including lifestyle factors (smoking, alcohol drinking, dietary factors, etc.) and medication use, were self-reported by participants, which may introduce information bias.

## Conclusions

In this large population-based study of individuals with SLD, we found that CMRF burden, measured by simple CMRF count, was strongly associated with increased risks of cardiovascular disease and mortality. While data-driven CMRF patterns revealed further heterogeneity in risk profiles, they did not offer substantial improvements in outcome prediction beyond CMRF count. These findings suggest that CMRF count remains a practical, informative and reliable measure for assessing cardiometabolic burden and stratifying cardiovascular risks in SLD population.

## Supplementary Information

Below is the link to the electronic supplementary material.

Supplementary Material 1.

## Data Availability

UK Biobank data are available to registered researchers at [https://www.ukbiobank.ac.uk/](https:/www.ukbiobank.ac.uk) .

## Abbreviations

SLD: steatotic liver disease
CMRF: cardiometabolic risk factors
CVD: cardiovascular disease
MASLD: metabolic dysfunction-associated steatotic liver disease
MetALD: metabolic-alcohol-related liver disease
ALD: alcohol-related liver disease
MRI: magnetic resonance imaging
PDFF: proton density fat fraction
BMI: body mass index
HbA1c: glycated haemoglobin
BP: blood pressure
TG: triglycerides
HDL: high-density lipoprotein
LCA: latent class analysis
BIC: Bayesian Information Criterion
aBIC: adjusted Bayesian Information Criterion
ICD-10: International Classification of Diseases, 10th version
HR: hazard ratio
CI: confidence interval
