# Supplementary material for: Measures of comorbid cardiometabolic burden and cardiovascular disease risk in people with MRI-confirmed steatotic liver disease: a prospective cohort study
Source: Cardiovasc Diabetol. 2026 Jan 29;25:64. doi: 10.1186/s12933-026-03088-1 (PMC12924323; doi:10.1186/s12933-026-03088-1)
Supplement: Supplementary file 1 — Supplementary Material 1. [file 12933_2026_3088_MOESM1_ESM.docx]

Measures of comorbid cardiometabolic burden and cardiovascular disease risk in people with MRI-confirmed steatotic liver disease: a prospective cohort study

Supplementary figure 1: Adjusted BIC versus number of clusters in latent class analysis.

BIC: Bayesian information criterion. Adjustment was for sample size.

Supplementary table 1: Baseline characteristics of participants, stratified by cardiometabolic risk factor counts.

|  | SLD | 1 | 2 | 3 | 4 | 5 |
| --- | --- | --- | --- | --- | --- | --- |
|  | n = 10121 | n = 525  (5.2%) | n = 2636 (26.1%) | n = 3585  (25.4%) | N = 2565 (25.3%) | n = 810  (8.0%) |
| Sex, male | 6163 (60.9%) | 303 (57.7%) | 1601 (60.7%) | 2493 (69.5%) | 1439 (56.1%) | 327 (40.4%) |
| Age, years | 64.7 (7.4) | 63.0 (7.7) | 63.9 (7.5) | 64.6 (7.5) | 65.4 (7.3) | 67.0 (6.9) |
| Townsend Deprivation Index |  |  |  |  |  |  |
| 1st fifth (least deprived) | 1929 (19.1%) | 112 (21.3%) | 523 (19.8%) | 689 (19.2%) | 474 (18.5%) | 131 (16.2%) |
| 5th fifth (most deprived) | 2154 (21.3%) | 98 (18.7%) | 517 (19.6%) | 739 (20.6%) | 601 (23.4%) | 199 (24.6%) |
| Education, higher education | 4274 (42.2%) | 272 (51.8%) | 1170 (44.4%) | 1514 (42.2%) | 1017 (39.6%) | 301 (37.2%) |
| Ethnicity, White | 9792 (96.7%) | 506 (96.4%) | 2551 (96.8%) | 3483 (97.2%) | 2483 (96.8%) | 769 (94.9%) |
| Smoking, never | 5881 (58.1%) | 354 (67.4%) | 1584 (60.1%) | 2086 (58.2%) | 1420 (55.4%) | 437 (54%) |
| Alcohol drinking, g/d* | 11.5 (2.6, 25.1) | 13.9 (4.7, 29.2) | 13.8 (4.4, 28.6) | 13.7 (3.4, 27.2) | 8.2 (1.5, 20.6) | 5.8 (0.8, 14.8) |
| Physical activity, high | 3324 (32.8%) | 190 (36.2%) | 936 (35.5%) | 1213 (33.8%) | 759 (29.6%) | 226 (27.9%) |
| BMI, kg/m2 | 29.7 (4.4) | 25.7 (3.6) | 28.8 (4.1) | 29.8 (4.1) | 30.6 (4.4) | 31.4 (4.8) |
| Waist circumference, cm | 97.7 (11.2) | 87.5 (9.2) | 95.4 (10.5) | 98.7 (10.6) | 99.8 (11.2) | 100.9 (11.6) |
| Liver fat, % | 10.8 (6.1) | 8.6 (4.6) | 9.8 (5.4) | 10.8 (6.2) | 11.7 (6.5) | 12.4 (6.8) |
| Systolic blood pressure, mmHg | 144.3 (17.4) | 132.7 (17.7) | 143.0 (17.5) | 145.9 (17.0) | 145.8 (16.9) | 144.7 (17.1) |
| Diastolic blood pressure, mmHg | 82.1 (9.7) | 77.5 (8.8) | 82.3 (9.8) | 83.1 (9.6) | 82.0 (9.7) | 79.7 (9.4) |
| Hypertension | 9326 (92.1%) | 271 (51.6%) | 2295 (87.1%) | 3419 (95.4%) | 2531 (98.7%) | 810 (100%) |
| Overweight/obesity | 9287 (91.8%) | 214 (40.8%) | 2303 (87.4%) | 3421 (95.4%) | 2539 (99%) | 810 (100%) |
| Prediabetes/diabetes | 2256 (22.3%) | 2 (0.4%) | 68 (2.6%) | 483 (13.5%) | 893 (34.8%) | 810 (100%) |
| High triglycerides | 5929 (58.6%) | 17 (3.2%) | 384 (14.6%) | 2388 (66.6%) | 2330 (90.8%) | 810 (100%) |
| Low HDL-cholesterol | 4064 (40.2%) | 21 (4%) | 222 (8.4%) | 1044 (29.1%) | 1967 (76.7%) | 810 (100%) |
| SLD subtypes |  |  |  |  |  |  |
| MASLD | 7791 (77.0%) | 367 (69.9%) | 1917 (72.7%) | 2715 (75.7%) | 2090 (81.5%) | 702 (86.7%) |
| MetALD | 1838 (18.2%) | 130 (24.8%) | 566 (21.5%) | 673 (18.8%) | 379 (14.8%) | 90 (11.1%) |
| ALD | 492 (4.9%) | 28 (5.3%) | 153 (5.8%) | 197 (5.5%) | 96 (3.7%) | 18 (2.2%) |

The table shows count (percent) for categorical variables and mean (standard deviation) for continuous variable, unless specially marked. *: showing median (interquartile interval). All p values < 0.01 for differences between the three groups, except for ethnicity (p = 0.38). P values were derived from chi-square test for categorical variables, analysis of variance test for normally distributed continuous variables, and Kruskal-Wallis test for other continuous variables. BMI: body mass index. HDL: high density lipoprotein. SLD: steatotic liver disease. MASLD: metabolic dysfunction associated steatotic liver disease. MetALD: metabolic and alcohol related liver disease. ALD: alcohol related liver disease.

Supplementary table 2: Associations (HR and 95%CI) between cardiometabolic risk factor count and the outcomes.

|  | 1 | 2 | | 3 | | 4 | 5 |
| --- | --- | --- | --- | --- | --- | --- | --- |
| Incidence |  | |  | |  |  |  |
| CVD | 5 / 516 | | 55 / 2595 | | 103 / 3494 | 74 / 2454 | 31 / 762 |
|  | Reference | | 1.93 (0.77, 4.83) | | 2.39 (0.97, 5.90) | 2.59 (1.04, 6.46) | 3.58 (1.36, 9.43) |
| MI | 3 / 516 | | 42 / 2595 | | 65 / 3494 | 50 / 2454 | 24 / 762 |
|  | Reference | | 2.31 (0.71, 7.46) | | 2.29 (0.72, 7.31) | 2.62 (0.81, 8.45) | 3.95 (1.16, 13.47) |
| Stroke | 2 / 516 | | 13 / 2595 | | 39 / 3494 | 24 / 2454 | 7 / 762 |
|  | Reference | | 1.30 (0.29, 5.78) | | 2.73 (0.66, 11.38) | 2.65 (0.62, 11.37) | 2.81 (0.56, 14.08) |
| Mortality |  |  | |  | |  |  |
| All-cause | 6 / 525 | 40 / 2636 | | 84 / 3585 | | 63 / 2565 | 19 / 810 |
|  | Reference | 1.15 (0.49, 2.72) | | 1.68 (0.73, 3.86) | | 1.96 (0.84, 4.57) | 2.20 (0.86, 5.62) |
| CVD * | 0 / 525 | 5 / 2636 | | 19 / 3585 | | 18 / 2565 | 4 / 810 |
|  | 0 | 0.38 | | 1.07 | | 1.42 | 1.01 |

CVD: cardiovascular disease. MI: myocardial infarction. The Cox model was stratified by age group, adjusted for sex, ethnicity, education, Townsend Deprivation Index (fifths), physical activity level, average daily alcohol drinking, smoking status, diet (vegetable, processed meat, and red meat) and medication uses (antihypertensives, statins, and antidiabetics). *: HR inestimable due to zero event in the reference group (people with 1 risk factor), therefore showing mortality rate (per 1000 person-years).

| (A) Lean SLD | (B) Overweight SLD |
| --- | --- |
|  |  |
| (C) Diabetic SLD | (D) CMRF count |
|  |  |

Supplementary figure 2: Characteristics of the predefined cardiometabolic risk factor patterns.

SLD: steatotic liver disease. TG: triglycerides. HDL: high density lipoprotein cholesterol. CMRF; cardiometabolic risk factor.

Supplementary table 3: Baseline characteristics of participants stratified by pre-defined cardiometabolic risk factor patterns

|  | SLD | Lean SLD | Overweight SLD | Diabetic SLD |
| --- | --- | --- | --- | --- |
|  | n = 10121 | n = 692  (6.8%) | N = 7173  (70.9%) | n = 2256 (22.3%) |
| Sex, male | 6163 (60.9%) | 496 (71.7%) | 4284 (59.7%) | 1383 (61.3%) |
| Age, years | 64.7 (7.4) | 65.4 (7.5) | 64.0 (7.5) | 66.7 (6.9) |
| Townsend Deprivation Index |  |  |  |  |
| 1st fifth (least deprived) | 1929 (19.1%) | 176 (25.4%) | 1344 (18.7%) | 409 (18.1%) |
| 5th fifth (most deprived) | 2154 (21.3%) | 128 (18.5%) | 1488 (20.7%) | 538 (23.8%) |
| Education, higher education | 4274 (42.2%) | 361 (52.2%) | 3050 (42.5%) | 863 (38.3%) |
| Ethnicity, White | 9792 (96.7%) | 660 (95.4%) | 7014 (97.8%) | 2118 (93.9%) |
| Smoking, never | 5881 (58.1%) | 453 (65.5%) | 4225 (58.9%) | 1203 (53.3%) |
| Alcohol drinking, g/d* | 11.5 (2.6, 25.1) | 13.9 (5.14, 26.9) | 12.1 (3.1, 26.3) | 8.6 (1.4, 20.9) |
| Physical activity, high | 3324 (32.8%) | 281 (40.6%) | 2357 (32.9%) | 686 (30.4%) |
| BMI, kg/m2 | 29.7 (4.4) | 23.6 (1.2) | 30.0 (3.9) | 30.5 (4.8) |
| Waist circumference, cm | 97.7 (11.2) | 83.9 (6.4) | 98.2 (10.3) | 100.3 (12.1) |
| Systolic blood pressure, mmHg | 144.3 (17.4) | 143.8 (17.7) | 144.1 (17.4) | 144.9 (17.4) |
| Diastolic blood pressure, mmHg | 82.1 (9.7) | 80.9 (9.8) | 82.7 (9.7) | 80.5 (9.6) |
| Hypertension | 9326 (92.1%) | 635 (91.8%) | 6526 (91.0%) | 2165 (96.0%) |
| Obesity | 9287 (91.8%) | 0 (0%) | 7173 (100.0%) | 2114 (93.7%) |
| Diabetes | 2256 (22.3%) | 0 (0%) | 0 (0%) | 2256 (100.0%) |
| High triglycerides | 5929 (58.6%) | 322 (46.5%) | 4072 (56.8%) | 1535 (68.0%) |
| Low HDL-cholesterol | 4064 (40.2%) | 214 (30.9%) | 2711 (37.8%) | 1139 (50.5%) |
| SLD subtypes |  |  |  |  |
| MASLD | 7791 (77.0%) | 516 (74.6%) | 5414 (75.5%) | 1861 (82.5%) |
| MetALD | 1838 (18.2%) | 134 (19.4%) | 1387 (19.3%) | 317 (14.1%) |
| ALD | 492 (4.9%) | 42 (6.1%) | 372 (5.2%) | 78 (3.5%) |

The table shows count(percent) for categorical variables and mean(standard deviation) for continuous variable, unless specially marked. *: showing median (interquartile interval). All p values for differences between the three groups < 0.01. P values were derived from chi-square test for categorical variables, analysis of variance test for normally distributed continuous variables, and Kruskal-Wallis test for other continuous variables. BMI: body mass index. HDL: high density lipoprotein. SLD: steatotic liver disease. MASLD: metabolic dysfunction associated steatotic liver disease. MetALD: metabolic and alcohol related liver disease. ALD: alcohol related liver disease.

Supplementary table 4: Associations between predefined cardiometabolic risk factor patterns and the outcomes

|  | Predefined cardiometabolic risk factor patterns | | |
| --- | --- | --- | --- |
|  | Lean SLD | Overweight SLD | Diabetic SLD |
| **Incidence** |  |  |  |
| **CVD** | 15 / 674 | 167 / 7002 | 86 / 2145 |
| Model 1 | Reference | 1.17 (0.69, 1.99) | 1.68 (0.97, 2.91) |
| Model 2 | Reference | 1.14 (0.67, 1.94) | 1.56 (0.89, 2.72) |
| Model 3 | Reference | 0.93 (0.53, 1.64) | 1.06 (0.55, 2.07) |
| **MI** | 9 / 674 | 115 / 7002 | 60 / 2145 |
| Model 1 | Reference | 1.35 (0.68, 2.66) | 1.94 (0.96, 3.91) |
| Model 2 | Reference | 1.26 (0.63, 2.49) | 1.65 (0.81, 3.37) |
| Model 3 | Reference | 1.08 (0.53, 2.23) | 1.24 (0.54, 2.87) |
| **Stroke** | 6 / 674 | 53 / 7002 | 26 / 2145 |
| Model 1 | Reference | 0.93 (0.40, 2.17) | 1.28 (0.53, 3.11) |
| Model 2 | Reference | 1.00 (0.43, 2.36) | 1.45 (0.59, 3.57) |
| Model 3 | Reference | 0.74 (0.30, 1.86) | 0.81 (0.27, 2.45) |
| **Mortality** |  |  |  |
| **All-cause** | 12 / 692 | 128 / 7173 | 72 / 2256 |
| Model 1 | Reference | 1.13 (0.62, 2.04) | 1.71 (0.93, 3.15) |
| Model 2 | Reference | 1.24 (0.67, 2.31) | 1.94 (1.02, 3.70) |
| Model 3 | Reference | 1.07 (0.55, 2.06) | 1.45 (0.68, 3.10) |
| **CVD** | 2 / 692 | 29 / 7173 | 15 / 2256 |
| Model 1 | Reference | 1.55 (0.37, 6.49) | 2.10 (0.48, 9.19) |
| Model 2 | Reference | 1.52 (0.36, 6.43) | 1.92 (0.43, 8.52) |
| Model 3 | Reference | 0.88 (0.19, 4.05) | 0.67 (0.12, 3.92) |

SLD: steatotic liver disease. CVD: cardiovascular disease. MI: myocardial infarction. Model 1: the cox model was unadjusted. Model 2: the Cox model was stratified by age group, adjusted for sex, ethnicity, education, Townsend Deprivation Index (fifths), physical activity level, average daily alcohol drinking, smoking status, diet (vegetable, processed meat, and red meat) and medication uses (antihypertensives, statins, and antidiabetics). Model 3: model 2 + CMRF count.

Supplementary table 5: Associations between cardiometabolic risk factor patterns with cardiovascular mortality and risks in Fine-Gray models adjusting for competing risk

|  |  | Latent class analysis derived cardiometabolic risk factor patterns | | |
| --- | --- | --- | --- | --- |
|  |  | Pattern A | Pattern B | Pattern C |
| Incidence |  |  |  |  |
| CVD | 268 / 9821 | 105 / 3216 | 156 / 5855 | 7 / 750 |
| Model 1 | 1.25 (1.12, 1.40) | Reference | 0.82 (0.64, 1.06) | 0.26 (0.13, 0.55) |
| Model 2 | 1.23 (1.09, 1.38) | Reference | 0.80 (0.63, 1.03) | 0.41 (0.16, 1.06) |
| Model 3 | -- | Reference | 1.23 (0.75, 2.02) | 0.74 (0.25, 2.17) |
| MI | 184 / 9821 | 74 / 3216 | 106 / 5855 | 4 / 750 |
| Model 1 | 1.26 (1.10, 1.44) | Reference | 0.80 (0.59, 1.06) | 0.22 (0.02, 2.59) |
| Model 2 | 1.19 (1.02, 1.39) | Reference | 0.81 (0.60, 1.09) | 0.39 (0.03, 4.91) |
| Model 3 | -- | Reference | 1.07 (0.60, 1.90) | 0.57 (0.04, 7.60) |
| Stroke | 85 / 9821 | 31 / 3216 | 51 / 5855 | 3 / 750 |
| Model 1 | 1.23 (1.01, 1.49) | Reference | 0.91 (0.56, 1.49) | 0.38 (0.01, 13.08) |
| Model 2 | 1.30 (1.07, 1.58) | Reference | 0.81 (0.49, 1.33) | 0.46 (0.00, 56.38) |
| Model 3 | -- | Reference | 1.70 (0.66, 4.38) | 1.26 (0.01, 180.30) |
| Mortality |  |  |  |  |
| CVD | 46 / 10121 | 22 / 3375 | 24 / 5985 | 0 / 761 |
| Model 1 | 1.49 (1.16, 1.90) | Reference | 0.62 (0.32, 1.19) | NA |
| Model 2 | 1.45 (1.12, 1.89) | Reference | 0.59 (0.33, 1.07) | NA |
| Model 3 | -- | Reference | 1.02 (0.34, 3.04) | NA |

CVD: cardiovascular disease. MI: myocardial infarction. Model 1: the model was unadjusted. Model 2: the model was adjusted for age, sex, ethnicity, education, Townsend Deprivation Index (fifths), physical activity level, average daily alcohol drinking, smoking status, diet (vegetable, processed meat, and red meat) and medication uses (antihypertensives, statins, and antidiabetics). Model 3: model 2 + CMRF count.

Supplementary table 6: Associations between cardiometabolic risk factor patterns with all-cause mortality, cardiovascular mortality and risks in people with MASLD

|  |  | Latent class analysis derived cardiometabolic risk factor patterns | | |
| --- | --- | --- | --- | --- |
|  | Per 1 more risk factor | Pattern A | Pattern B | Pattern C |
| Incidence |  |  |  |  |
| CVD | 207 / 7557 | 86 / 2664 | 117 / 4284 | 4 / 609 |
|  | 1.21 (1.05, 1.41) | Reference | 1.39 (0.82, 2.37) | 0.62 (0.19, 1.99) |
| MI | 141 / 7557 | 62 / 2664 | 77 / 4284 | 2 / 609 |
|  | 1.19 (0.99, 1.42) | Reference | 1.03 (0.54, 1.95) | 0.35 (0.07, 1.75) |
| Stroke | 67 / 7557 | 24 / 2664 | 41 / 4284 | 2 / 609 |
|  | 1.26 (0.97, 1.64) | Reference | 2.74 (1.05, 7.13) | 1.74 (0.29, 10.44) |
| Mortality |  |  |  |  |
| All-cause | 162 / 7791 | 65 / 2792 | 86 / 4381 | 11 / 618 |
|  | 1.22 (1.03, 1.43) | Reference | 1.46 (0.80, 2.65) | 2.34 (0.94, 5.87) |
| CVD | 38 / 7791 | 18 / 2792 | 20 / 4381 | 0 / 618 |
|  | 1.30 (0.91, 1.85) | Reference | 1.07 (0.30, 3.78) | NA |

The Cox model was stratified by age group, adjusted for sex, ethnicity, education, Townsend Deprivation Index (fifths), physical activity level, average daily alcohol drinking, smoking status, diet (vegetable, processed meat, and red meat) and medication uses (antihypertensives, statins, and antidiabetics), and CMRF count.

Supplementary table 7: Associations between cardiometabolic risk factor patterns with all-cause mortality, cardiovascular mortality and risks in people with MetALD

|  |  | Latent class analysis derived cardiometabolic risk factor patterns | | |
| --- | --- | --- | --- | --- |
|  | Per 1 more risk factor | Pattern A | Pattern B | Pattern C |
| Incidence |  |  |  |  |
| CVD | 46 / 1790 | 13 / 446 | 30 / 1219 | 3 / 125 |
|  | 1.24 (0.89, 1.74) | Reference | 0.88 (0.29, 2.61) | 1.30 (0.21, 8.15) |
| MI | 33 / 1790 | 10 / 446 | 21 / 1219 | 2 / 125 |
|  | 1.27 (0.86, 1.89) | Reference | 1.07 (0.29, 3.92) | 1.94 (0.21, 18.09) |
| Stroke | 13 / 1790 | 3 / 446 | 9 / 1219 | 1 / 125 |
|  | 1.13 (0.61, 2.08) | Reference | 0.80 (0.10, 6.49) | 0.88 (0.03, 22.65) |
| Mortality |  |  |  |  |
| All-cause | 30 / 1838 | 10 / 469 | 17 / 1242 | 3 / 127 |
|  | 1.16 (0.77, 1.74) | Reference | 0.60 (0.15, 2.38) | 2.09 (0.25, 17.75) |
| CVD | 6 / 1838 | 3 / 469 | 3 / 1242 | 0 / 127 |
|  | 1.87 (0.56, 6.24) | Reference | 1.15 (0.03, 45.80) | NA |

The Cox model was stratified by age group, adjusted for sex, ethnicity, education, Townsend Deprivation Index (fifths), physical activity level, average daily alcohol drinking, smoking status, diet (vegetable, processed meat, and red meat) and medication uses (antihypertensives, statins, and antidiabetics), and CMRF count.

Supplementary table 8: Sex-specific associations between cardiometabolic risk factor count and patterns with all-cause mortality, cardiovascular mortality and risk.

|  |  | Predefined CMRF patterns | | | Derived CMRF clusters | | |
| --- | --- | --- | --- | --- | --- | --- | --- |
|  | Per 1 more risk factor | Lean SLD | Overweight SLD | Diabetic SLD | Pattern A | Pattern B | Pattern C |
| Males |  |  |  |  |  |  |  |
| Incidence |  |  |  |  |  |  |  |
| CVD | 199 / 5916 | 12 / 482 | 128 / 4141 | 59 / 1293 | 66 / 1647 | 128 / 3943 | 5 / 326 |
|  | 1.17 (1.01, 1.36) | Reference | 1.21 (0.66, 2.20) | 1.52 (0.81, 2.86) | Reference | 0.85 (0.63, 1.15) | 0.48 (0.19, 1.19) |
| MI | 141 / 5916 | 7 / 482 | 91 / 4141 | 43 / 1293 | 49 / 1647 | 88 / 3943 | 4 / 326 |
|  | 1.16 (0.97, 1.39) | Reference | 1.40 (0.65, 3.05) | 1.78 (0.79, 4.00) | Reference | 0.80 (0.56, 1.15) | 0.52 (0.19, 1.45) |
| Stroke | 58 / 5916 | 5 / 482 | 37 / 4141 | 16 / 1293 | 17 / 1647 | 40 / 3943 | 1 / 326 |
|  | 1.20 (0.91, 1.58) | Reference | 0.95 (0.37, 2.44) | 1.15 (0.41, 3.20) | Reference | 0.99 (0.56, 1.76) | 0.37 (0.05, 2.79) |
| Mortality |  |  |  |  |  |  |  |
| All-cause | 162 / 6163 | 8 / 496 | 93 / 4284 | 61 / 1383 | 64 / 1766 | 93 / 4062 | 5 / 335 |
|  | 1.31 (1.11, 1.54) | Reference | 1.49 (0.69, 3.23) | 2.44 (1.11, 5.39) | Reference | 0.67 (0.48, 0.92) | 0.61 (0.24, 1.53) |
| CVD | 40 / 6163 | 2 / 496 | 25 / 4284 | 13 / 1383 | 17 / 1766 | 23 / 4062 | 0 / 335 |
|  | 1.42 (1.01, 1.99) | Reference | 1.41 (0.33, 6.04) | 1.75 (0.39, 7.89) | Reference | 0.64 (0.33, 1.22) | NA |
| Females |  |  |  |  |  |  |  |
| Incidence |  |  |  |  |  |  |  |
| CVD | 69 / 3905 | 3 / 192 | 39 / 2861 | 27 / 852 | 39 / 1569 | 28 / 1912 | 2 / 424 |
|  | 1.42 (1.08, 1.87) | Reference | 0.90 (0.27, 2.94) | 1.55 (0.46, 5.23) | Reference | 0.64 (0.36, 1.15) | 0.30 (0.07, 1.33) |
| MI | 43 / 3905 | 2 / 192 | 24 / 2861 | 17 / 852 | 25 / 1569 | 18 / 1912 | 0 / 424 |
|  | 1.35 (0.94, 1.92) | Reference | 0.82 (0.19, 3.56) | 1.23 (0.27, 5.50) | Reference | 0.84 (0.39, 1.83) | NA |
| Stroke | 27 / 3905 | 1 / 192 | 16 / 2861 | 10 / 852 | 14 / 1569 | 11 / 1912 | 2 / 424 |
|  | 1.56 (1.01, 2.41) | Reference | 1.20 (0.16, 9.24) | 2.57 (0.32, 20.96) | Reference | 0.47 (0.19, 1.15) | 0.47 (0.10, 2.22) |
| Mortality |  |  |  |  |  |  |  |
| All-cause | 50 / 3958 | 4 / 196 | 35 / 2889 | 11 / 873 | 18 / 1609 | 22 / 1923 | 10 / 426 |
|  | 1.05 (0.77, 1.43) | Reference | 0.81 (0.28, 2.38) | 0.90 (0.27, 3.00) | Reference | 0.87 (0.41, 1.84) | 2.30 (0.93, 5.68) |
| CVD | 6 / 3958 | 0 / 196 | 4 / 2889 | 2 / 873 | 5 / 1609 | 1 / 1923 | 0 / 426 |
|  | 2.05 (0.66, 6.33) | Reference | NA | NA | Reference | 0.20 (0.02, 2.29) | NA |

SLD: steatotic liver disease. CVD: cardiovascular disease. MI: myocardial infarction. The Cox model was stratified by age group, adjusted for ethnicity, education, Townsend Deprivation Index (fifths), physical activity level, average daily alcohol drinking, and smoking status, diet (vegetable, processed meat, and red meat) and medication uses (antihypertensives, statins, and antidiabetics).

Supplementary table 9: Associations between individual cardiometabolic risk factors and all-cause mortality, cardiovascular mortality and risks

|  | Obesity | Hypertension | Diabetes | High TG | Low HDL |
| --- | --- | --- | --- | --- | --- |
| Incidence |  |  |  |  |  |
| CVD | 0.99 (0.64, 1.54) | 2.11 (0.99, 4.52) | 1.35 (1.03, 1.76) | 1.19 (0.92, 1.55) | 1.15 (0.88, 1.50) |
| MI | 1.02 (0.59, 1.74) | 2.26 (0.83, 6.17) | 1.32 (0.96, 1.81) | 1.13 (0.83, 1.55) | 1.11 (0.81, 1.54) |
| Stroke | 0.98 (0.46, 2.06) | 1.91 (0.59, 6.15) | 1.39 (0.87, 2.25) | 1.37 (0.86, 2.18) | 1.20 (0.75, 1.90) |
| Mortality |  |  |  |  |  |
| All-cause | 1.18 (0.71, 1.98) | 0.72 (0.42, 1.25) | 1.56 (1.16, 2.09) | 1.34 (1.01, 1.80) | 1.08 (0.80, 1.45) |
| CVD | 2.02 (0.48, 8.47) | NA | 1.22 (0.65, 2.31) | 1.43 (0.74, 2.78) | 1.45 (0.77, 2.71) |

The Cox model was stratified by age group, adjusted for sex, ethnicity, education, Townsend Deprivation Index (fifths), physical activity level, average daily alcohol drinking, smoking status, diet (vegetable, processed meat, and red meat) and medication uses (antihypertensives, statins, and antidiabetics).
